# Association Between Low Kidney Function and Excess Weight Concerning Unfavourable Periodontal Health among Community-dwelling Older Japanese Women

**DOI:** 10.3290/j.ohpd.b5573943

**Published:** 2024-07-23

**Authors:** Akihiro Yoshihara, Masanori Iwasaki, Kana Suwama, Kazutoshi Nakamura

**Affiliations:** a Professor, Division of Oral Science for Health Promotion, Faculty of Dentistry & Graduate School of Medical and Dental Sciences, Niigata University, Niigata, Japan. Study concept, analysis and interpretation, drafted the article, significant intellectual content review.; b Professor, Division of Preventive Dentistry, Graduate School of Dental Medicine, Hokkaido University, Hokkaido, Japan. Study concept, analysis and interpretation, data collection and compilation, significant intellectual content review.; c Assistant Professor, Division of Oral Science for Health Promotion, Faculty of Dentistry & Graduate School of Medical and Dental Sciences, Niigata University, Niigata, Japan. Analysis and interpretation, substantial intellectual content review.; d Professor, Department of Community Preventive Medicine, Niigata University Graduate School of Medical and Dental Sciences, Niigata, Japan. Study concept, data collection and compilation analysis and interpretation, significant intellectual content review.

**Keywords:** excess weight, kidney function, periodontal health

## Abstract

**Purpose:**

To investigate the association of low renal function and overweight with poor periodontal condition in community-dwelling older Japanese women.

**Materials and Methods:**

In total, 359 older women (age range: 55–74 years) participated in this study. Two periodontal parameters – the number of teeth with a probing pocket depth (PPD) or clinical attachment level (CAL) ≥ 4 mm – were used as the dependent variables. The principal independent variables were low renal function as defined by the estimated glomerular filtration rate (eGFR) and overweight as defined by the body mass index. Poisson regression analysis was used to calculate the ratio of means (RM).

**Results:**

The RMs of the number of teeth with a PPD or CAL ≥ 4 mm in an adjusted model without an interaction term were 1.21- or 1.27-fold higher among those with an eGFR < 60, while those among the participants with an eGFR < 60 in the adjusted model with interaction terms for the number of teeth with a PPD or CAL ≥ 4 mm were 1.43- or 1.36-fold higher. In addition, increments of periodontal risk with low renal function and overweight showed a slightly smaller to negative trend.

**Conclusion:**

The present findings suggest a connection between unfavourable periodontal health and both renal function and being overweight among older Japanese women. A weak negative interaction was also found between poor renal condition and overweight in relation to periodontal condition.

Periodontal disease is a well-known cause of infectious inflammation. Earlier studies have proposed a link between periodontal disease and diminished renal function. Iwasaki et al^16^ reported that periodontal disease is related to chronic kidney disease (CKD), which is usually marked by albuminuria or a decreased glomerular filtration rate (GFR). Frequent origins of CKD encompass persistent hypertension, diabetes mellitus (DM), and glomerulonephritis.^32^ Furthermore, patients undergoing dialysis have been shown to have relatively worse cases of periodontal disease compared with healthy adults,^5^ and a significant relationship has been reported between diminished kidney function and tooth loss.^35^ In a longitudinal study, an association was reported between CKD and poor periodontal conditions.^17^ Chronic low-level inflammation might be a key factor connecting these conditions, suggesting that periodontal disease could be an underlying comorbidity of chronic kidney disease.^27^ While periodontal disease and chronic kidney disease (CKD) are intricate conditions with shared behavioural, social, and other confounding factors, there might also be an independent association between the two conditions.^14^ Therefore, we considered whether poor renal function might lead to a deteriorated periodontal condition not only in patients undergoing dialysis, but also in patients with CKD not undergoing dialysis.

On the other hand, an association has also been reported between obesity and periodontal disease,^29^ which are the most commonly occurring noncommunicable diseases, and statistically significant associations have been found between obesity and both the presence and severity of periodontal conditions.^24^ The interaction between periodontal disease, diabetes, and obesity is supported by mutual biological mechanisms, including systemic inflammation, insulin resistance, and metabolic dysfunction, alongside shared risk factors related to lifestyle.^21^

Being overweight is the primary factor that initiates DM, which is marked by a disrupted systemic inflammatory condition induced by an uneven cytokine network involving acute-phase proteins and proinflammatory cytokines.^25^ The immune function of adipose tissue is believed to have significant functions in not only insulin resistance, but also the initiation and advancement of periodontal disease. In addition, a link has been suggested between reduced renal function, an unfavourable periodontal status, and being overweight.^38^ However, to the authors’ knowledge to date, no studies have examined this connection. From recent studies, it is suggested that common mediators such as NT-PRO-BNP and TGF beta1 may play a role in influencing periodontal disease, obesity, and chronic kidney disease.^1^^,^^6^

Therefore, we considered the possibility of an interaction between poor periodontal condition, low renal function, and overweight. Elucidating this relationship could offer valuable contextual insights for managing periodontal disease among individuals with low renal function.

Building upon this foundation, the present study was aimed to investigate the association of low renal function and overweight with poor periodontal condition in community-dwelling older Japanese women.

## MATERIALS AND METHODS

### Participants and Selection

**Fig 1:**
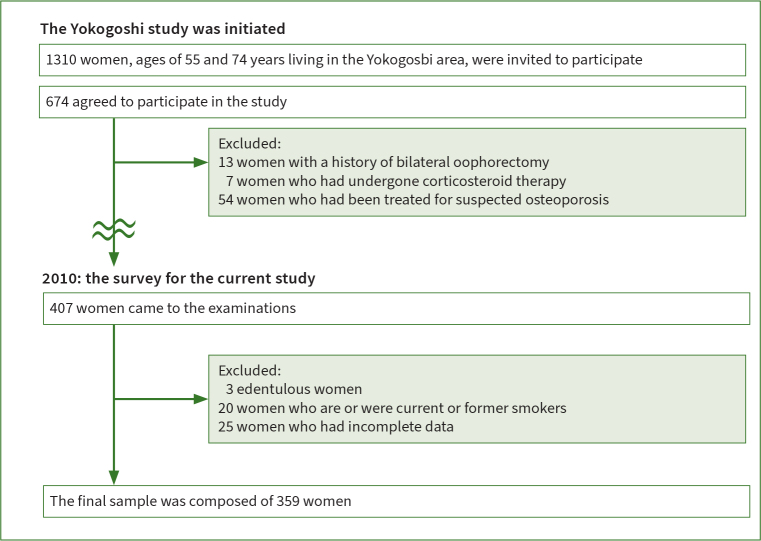
(Fig 1 Flow diagram of the present study.

The research design was a cross-sectional survey. We targeted all 1310 postmenopausal women aged 55-74 years who lived in the town of Yokogoshi, Japan.^26^ Among these residents, 674 (51.5%) provided written informed consent and willingly took part in the study. Because bone metabolism might affect periodontal disease,^33^^,^^34^^,^^37^ we excluded individuals with medical histories that could have impacted bone metabolism: (i) 13 individuals who had undergone bilateral oophorectomy; (ii) seven who had received corticosteroid therapy; (iii) 54 who had received treatment such as bisphosphonates, selective estrogen receptor modulators, active vitamin D analogs, vitamin K, estrogen, or calcitonin due to suspected osteoporosis. Further, we excluded (iv) three who were completely edentulous; and (v) 20 current or former smokers in personal interviews. In addition, (vi) 193 women were excluded because they did not participate in the 2010 examinations, and (vii) 25 were excluded due to insufficient data. The final study cohort comprised 359 women ((Fig 1).

First, blood samples were obtained at least 6 hours after a daytime meal and promptly stored at 4°C. On the same day, each sample underwent centrifugation at 1613xg for 10 min. The resultant serum was then stored at a temperature of -40°C until it was ready for biochemical analysis. Serum creatinine was used to assess renal function, while glycated hemoglobin (HbA1C; %) was used to examine serum markers associated with diabetes. Serum creatinine and HbA1C levels were both determined at a commercial laboratory in Japan (BML; Tokyo, Japan).

The Ethics Committee of Niigata University School of Medicine (approval No. H15-0078) approved this observational study involving human subjects. The research was carried out in compliance with the principles of the Declaration of Helsinki and all applicable STROBE guidelines.^20^

### Dependent Variables

In this study, the dependent variables consisted of two periodontal parameters: the number of teeth exhibiting a probing pocket depth (PPD) or a clinical attachment level (CAL) ≥ 4 mm. All oral assessments, including the periodontal evaluations, were performed by two proficient dentists in adequately illuminated conditions. Following the determination of the remaining tooth count, periodontal parameters such as PPD and CAL were assessed at the midbuccal and mesiobuccal locations of each tooth based on NHANES III.^2^ Next, the periodontal status of each tooth, including the third molars, was documented and rounded to the nearest whole millimeter. In situations where a restorative margin extended below the cementoenamel junction (CEJ), CAL was assessed considering the anatomical characteristics of the individual tooth and the CEJ of the neighbouring tooth/teeth, as applicable. Before the initiation of this study, both inter- and intra-observer agreement regarding PPD and CAL were assessed in patients who had previously undergone an examination at the Clinic of Preventive Dentistry at Niigata University Medical and Dental Hospital. When the difference was ≤ 1 mm, the measurements were considered to be in agreement. The inter- and intra-observer agreement for PPD ≥ 4 mm were 85.7% and 100%, and those for CAL ≥ 4 mm were 64.5% and 78.6%, respectively, for one examiner; for the other examiner, the intra-observer agreement was 85.7% for PPD ≥ 4 mm and 81.3% for CAL ≥ 4 mm.

### Independent Variables

Renal function and weight were the principal exposure variables in the present study. Low renal function was defined as an estimated GFR (eGFR) < 60 ml/min/1.73 m^2^, and overweight as a body mass index (BMI) ≥ 25 kg/m^2^. The following abbreviated “Modification of Diet in Renal Disease Study equation”, modified by a coefficient for Japanese people,^23^ was used to calculate the eGFR:

 eGFR (ml/min/1.73 m^2^) = 194 x age–0.287 x serum creatinine–1.094

(if female, x 0.739)

The height and weight of each participant were measured to calculate BMI. Age, serum HbA1C level, the number of teeth present, the use of interdental brushes or dental floss, and undergoing professional tooth cleaning (PTC) during the past year were obtained through participant interviews and were selected as confounding factors.

### Statistical Analysis

First, the mean values of selected characteristics were calculated for each quintile of the eGFR (mean ± SD, ml/min/1.73 m^2^ [1st: 58.4 ± 6.1; 2nd: 67.7 ± 1.5; 3rd: 74.9 ± 2.7; 4th: 83.4 ± 2.3; 5th: 96.1 ± 7.4]). The Jonckheere-Terpstra test, which is a test for trends across ordered groups, was performed. The independent variables were categorised as follows in the statistical models: eGFR (1: < 60, 0: ≥ 60) and BMI (0: < 25, 1: ≥ 25). Due to the skewed distribution of teeth with PPD or CAL ≥ 4 mm, following a Poisson distribution pattern, both univariate and multivariate Poisson regression analyses were performed. These analyses aimed to gauge the ratio of means (RM) concerning renal function or overweight. The RM represents the multiplicative change in the estimated mean of a dependent variable for a one-category escalation in the independent variable.^11^ Univariate analysis and an adjusted model without interaction terms, including all independent variables (age, serum HbA1C level, number of teeth present, the use of interdental brushes or dental floss, and undergoing PTC during the past year), were conducted. In addition, the interaction term between the eGFR and BMI was added in a fully adjusted model. Finally, we evaluated weighted RMs multiplied by the interaction terms for the periodontal parameters for BMI (< 25 or ≥ 25) and eGFR (< 60 or ≥ 60) values using the following formula:

 RM of interaction term x RM of eGFR (1: < 60, 0: ≥ 60) x RM of BMI (0: < 25, 1: ≥ 25)

All computations and statistical evaluations were conducted utilising STATA/SE 17.0 (StataCorp, College Station; TX, USA). A significance level of = 0.05 was adopted to determine statistical significance.

## RESULTS

Table 1 presents the demographic, laboratory, eGFR, and BMI characteristics of the participants investigated. In the study population, the mean eGFR (± SD) was 76.1 ± 13.7 ml/min/ 1.73 m^2^, while the mean BMI (± SD) was 22.7 ± 3.6 kg/m^2^. Correspondingly, the number of teeth exhibiting PPD and CAL ≥ 4 mm was summarised as [median (25th percentile/75th percentile)] 2 (1/4) and 4 (2/9).

**Table 1:** Selected characteristics of the study participants (n = 359)

Variables	
Age (mean ± SD, years)	63.9 ± 5.5
eGFR (mean ± SD, ml/min/1.73 m^2^)	76.1 ± 13.7
BMI (mean ± SD, kg/m^2^)	22.7 ± 3.6
Serum HbA1C levels (mean ± SD, %)	5.1 ± 0.5
Use of dental floss or interdental brush (yes, %)	37.6
Underwent PTC during the past year (yes, %)	31.5
Number of teeth present (mean ± SD)	23.4 ± 5.1
Number of sites with a PPD ≥4 mm [median (25th percentile /75th percentile)]	2 (1/4)
Number of sites with a CAL ≥4 mm [median (25th percentile/75th percentile)]	4 (2/9)

BMI: body mass index; PTC: professional tooth cleaning; BOP: bleeding on probing; CAL: clinical attachment level; PPD: probing pocket depth; SD: standard deviation.

Table 2 illustrates the descriptive connections among eGFR quintile clusters. The number of teeth demonstrating PPD or CAL ≥ 4 mm was summarised as [median (25th percentile/75th percentile)] of 2 (1/5.5) or 5.5 (2/9.5) for the 1st quintile and 1 (0/3) or 3 (2/8) for the 5th quintile. A negative trend was observed between eGFR quintile groups and the number of teeth exhibiting PPD or CAL ≥ 4 mm. The p-values for trends were 0.021 for the number of teeth with PPD ≥ 4 mm and 0.017 for the number of teeth with CAL ≥ 4 mm.

**Table 2:** Comparison of selected characteristics and eGFR

Variables	eGFR (ml/min/1.73 m^2^)					p-value*
	1st (n = 72)	2nd (n = 72)	3rd (n = 72)	4th (n = 72)	5th (n = 71)	
eGFR (mean ± SD, ml/min/1.73 m^2^)	58.4 ± 6.1	67.7 ± 1.5	74.9 ± 2.7	83.4 ± 2.3	96.1 ± 7.4	<0.001
BMI (mean ± SD, kg/m^2^)	22.8 ± 3.3	22.9 ± 4.1	23.1 ± 3.3	22.7 ± 3.7	22.2 ± 3.3	0.326
Age (mean ± SD, years)	66.4 ± 5.6	63.9 ± 5.	64.1 ± 5.2	62.9 ± 5.5	62.0 ± 5.0	<0.001
Serum HbA1C level (mean ± SD, %)	5.0 ± 0.4	5.1 ± 0.4	5.2 ± 0.5	5.1 ± 0.4	5.2 ± 0.7	0.396
Number of teeth present (mean ± SD)	22.6 ± 6.0	23.4 ± 4.9	22.6 ± 5.9	24.0 ± 4.3	24.2 ± 4.2	0.188
Number of teeth with PPD ≥4 mm [median (25th percentile/75th percentile)]	2 (1/5.5)	2 (1/5)	1 (1/4)	1.5 (1/3)	1 (0/3)	0.021
Number of teeth with CAL ≥4 mm [median (25th percentile/75th percentile)]	5.5 (2/9.5)	5.5 (2.5/10)	5 (2/9)	4 (2/8)	3 (2/8)	0.017

* Jonckheere–Terpstra test; BMI: body mass index; eGFR: estimated glomerular filtration rate; CAL: clinical attachment level; PPD: probing pocket depth; SD: standard deviation.

In Table 3, the RM for eGFR and BMI concerning the number of teeth exhibiting PPD or CAL ≥ 4 mm are presented across different analytical models. These include the univariate analysis model, an adjusted model considering covariates (such as the number of teeth present, age, serum HbA1C level, utilisation of interdental brushes or dental floss, and undergoing PTC during the previous year) without the inclusion of the interaction term. Additionally, the fully adjusted model considers covariates (number of teeth present, age, serum HbA1C level, utilisation of interdental brushes or dental floss, and undergoing PTC during the past year) while incorporating the interaction term.

**Table 3:** RMs and 95% confidence intervals for eGFR and BMI and the interaction term for periodontal condition markers

PPD				
Independent variables		Dependent variable		
		Number of teeth with a PPD ≥4 mm		
		Crude RM (95%CI)	Adjusted RM (95%CI)*	Fully adjusted RM (95%CI)**
eGFR	≥ 60 (H)	1.00 (reference)	1.00 (reference)	1.00 (reference
(ml/min/1.73 m^2^)				
	< 60 (L)	1.44 (1.22–1.71)	1.21 (1.01–1.44)	1.43 (1.16–1.76)
BMI	< 25.0 (L)	1.00 (reference)	1.00 (reference)	1.00 (reference)
(kg/m^2^)				
	≥ 25.0 (H)	1.07 (0.93–1.22)	1.12 (0.98–1.29)	1.22 (1.05–1.41)
Interaction term (eGFR x BMI)	eGFR (H) x BMI (L)			1.00 (reference)
	eGFR (L) x BMI (H)			0.59 (0.40–0.86)
**CAL**				
Independent variables		Dependent variable		
		Number of teeth with a PPD ≥4 mm		
		Crude RM (95%CI)	Adjusted RM (95%CI)*	Fully adjusted RM (95%CI)**
eGFR	≥60 (L)	1.00 (reference)	1.00 (reference)	1.00 (reference)
(ml/min/1.73 m^2^)				
	< 60 (H)	1.34 (1.18–1.51)	1.27 (1.11–1.44)	1.36 (1.17–1.58)
BMI	< 25.0 (L)	1.00 (reference)	1.00 (reference)	1.00 (reference)
(kg/m^2^)				
	≥ 25.0 (H)	0.95 (0.86–1.05)	0.96 (0.86–1.06)	0.99 (0.89–1.11)
Interaction term (eGFR x BMI)	eGFR (H) x BMI (L)			1.00 (reference)
	eGFR (L) x BMI (H)			0.80 (0.61–1.05)

*Adjusted by age, serum HbA1c, the number of teeth present, underwent professional teeth cleaning during the past year, and use of dental floss or an interdental brush. ** Adjusted by adding an interaction term. BMI: body mass index. eGFR: estimated glomerular filtration rate. CI: confidence interval. RM: ratio of means. CAL: clinical attachment level. PPD: probing pocket depth.

The primary outcomes revealed that among participants with an eGFR < 60 ml/min/1.73 m^2^, the adjusted RMs in the adjusted model (without the inclusion of the interaction term) for the number of teeth with PPD or CAL ≥ 4 mm were 1.21 and 1.27, respectively. Similarly, the adjusted RMs for BMI ≥ 25 kg/m^2^ in the adjusted model (without the interaction term) for the number of teeth with PPD and CAL ≥ 4 mm were 1.12 and 0.96, respectively. Neither of these values were statistically significance. Correspondingly, in the univariate analysis, individuals with an eGFR < 60 ml/min/1.73 m^2^ were more likely to exhibit a greater number of teeth with PPD or CAL ≥ 4 mm compared with those with an eGFR ≥ 60 ml/min/1.73 m^2^.

**Fig 2:**
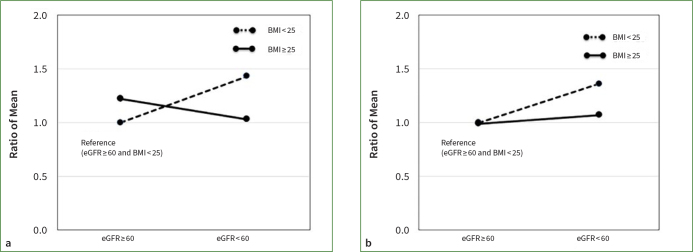
(Fig 2 Interaction plot between body mass index (BMI) and the estimated glomerular filtration rate (eGFR) for periodontal parameters. a) Probing pocket depth (PPD); b) clinical attachment level (CAL). Weighted ratio of means (RM) adjusted by the interaction term for periodontal parameters.

Furthermore, an examination of interaction effects demonstrated that the fully adjusted RM for the interaction term concerning the number of teeth with PPD ≥ 4 mm was 0.59. Similarly, the interaction term for the number of teeth with CAL ≥ 4 mm was 0.80; however, this difference was not statistically significant. The RMs associated with an eGFR < 60 ml/min/1.73 m^2^, considering the interaction term, were 1.43 and 1.36 for the number of teeth with PPD or CAL ≥ 4 mm, respectively. For BMI ≥ 25 kg/m^2^ with the interaction term, the corresponding RMs were 1.22 and 0.99 for the number of teeth with PPD and CAL ≥ 4 mm, respectively. The interaction plot, depicted in (Fig 2, illustrates the findings of subgroup analyses involving the interaction between eGFR (1: < 60 or 0: ≥ 60 ml/min/1.73 m^2^) and BMI (0: < 25 or 1: ≥ 25 kg/m^2^) values. In general, the RM for periodontal parameters showed an increase in participants with an eGFR < 60 ml/min/1.73 m^2^ and in those with a BMI ≥ 25 kg/m^2^ (as shown in Table 3). However, the augmentation of periodontal risk when both low renal function and overweight were present exhibited a slightly smaller to negative trend, as indicated by the RM of the interaction term, which was < 1.00 (as detailed in Table 3).

## DISCUSSION

In this study, the RMs associated with an eGFR < 60 ml/min/1.73 m^2^, considering the interaction term, were 1.43 and 1.36 for the number of teeth with PPD or CAL ≥ 4 mm, respectively. The findings of this study revealed an association between CKD and poor periodontal status in older Japanese women.

In a previous investigation, we also found that renal function might be influenced not only by inflamed periodontal tissues, but also by a wider state of inflammation.^36^ The association between CKD and periodontal disease has been documented, with systemic inflammation recognised as a contributory risk factor for CKD.^15^

Renal function plays a vital role in governing mineral metabolism,^19^ including the conversion of vitamin D into its active form, and diminished renal function has been shown to impede this conversion process.^19^ In the context of bone metabolism, vitamin D plays a role in regulating the release of hormones such as parathyroid hormone and overseeing the functioning of calcium phosphate.^8^ Additionally, it plays a key role in governing the production of bone matrix proteins, which are notably linked with periodontal parameters such as PPD and CAL.^8^^,^^34^ Consequently, the impact of low renal function on bone metabolism could potentially exert an effect on the state of periodontal health.

Furthermore, despite the lack of statistical significance (as indicated in Table 3), the RMs for periodontal indicators such as the number of teeth with PPD ≥ 4 mm and BMI ≥ 25 in the adjusted model were greater than 1.00. These results suggest a potential link between a high BMI and suboptimal periodontal health among older Japanese.

A bidirectional relationship has been proposed between periodontal disease and metabolic syndrome, based on circulating cytokines and oxidative stress.^8^ The higher RMs for poor periodontal condition among individuals with a BMI ≥ 25 observed in the present study supports those findings. According to a systematic review, identifying a plausible underlying mechanism could clarify the connection between being overweight and unfavourable periodontal health. One such mechanism involves the emergence of insulin resistance, which has been linked to both excess weight and a suboptimal periodontal state due to persistent inflammation and oxidative stress.^22^ In addition, several longitudinal cohort studies^10^^,^^12^ have documented a direct correlation between weight gain and the advancement of periodontal disease.

Within the scope of this study, an high BMI and low renal function demonstrated a notable association with a worse periodontal condition. However, according to the interaction plot ((Fig 2), people who are overweight and have low renal function are more likely to have a poorer periodontal condition. Moreover, a statistically significant interaction was found between overweight and low renal function; this does not simply derive from the addition of the risk for each factor, but does work in the direction of slightly lowering the risk.

Obesity is closely related to diabetes and hypertension, and overweight is a primary cause of CKD.^4^^,^^31^ However, some reports said that weight gain may have a positive effect. Links have been established between obesity and chronic noncommunicable diseases. For example, an elevated BMI has been recognised for its association with improved survival rates in individuals affected by conditions such as CKD, cardiovascular disease, and DM.^4^^,^^9^^,^^18^ In addition, according to previous studies, the role of periodontal inflammation as a hidden cause of heightened systemic oxidative stress in CKD is emphasised in the preceding studies. Furthermore, oxidative stress is a major factor in the pathophysiology of obesity. There is potential involvement of oxidative stress in the mechanisms connecting these.^28^^,^^30^ However, additional research is needed to elucidate the underlying mechanisms that link periodontal health, overweight, and renal function.

This study has several limitations. First, given its cross-sectional design, the associations observed between periodontal parameters and renal function or BMI could potentially involve a reverse causal relationship. Prior research has indicated that individuals with CKD tend to exhibit increased levels of plaque accumulation, calculus formation, and gingival inflammation, along with reduced salivary output, collectively suggesting an overall decline in oral hygiene.^7^ Regrettably, the present study lacked information concerning oral hygiene practices.

## CONCLUSION

The present findings imply an association between suboptimal periodontal health and both low renal function and overweight. Furthermore, a modest adverse interaction was identified between compromised renal function and excess weight concerning periodontal health. Consequently, when addressing periodontal disease in individuals with low renal function, it is advisable to formulate a treatment strategy while taking the patient’s BMI into account.
